# A Concept Analysis on the Use of Artificial Intelligence in Nursing

**DOI:** 10.7759/cureus.14857

**Published:** 2021-05-05

**Authors:** Zhida Shang

**Affiliations:** 1 Faculty of Medicine and Health Sciences, McGill University, Montreal, CAN

**Keywords:** nursing, artificial intelligence, ai, concept analysis, deep learning artificial intelligence

## Abstract

Artificial intelligence (AI) has a considerable present and future influence on healthcare. Nurses, representing the largest proportion of healthcare workers, are set to immensely benefit from this technology. However, the overall adoption of new technologies by nurses is quite slow, and the use of AI in nursing is considered to be in its infancy. The current literature on AI in nursing lacks conceptual clarity and consensus, which is affecting clinical practice, research activities, and theory development. Therefore, to set the foundations for nursing AI knowledge development, the purpose of this concept analysis is to clarify the conceptual components of AI in nursing and to determine its conceptual maturity. A concept analysis following Morse’s approach was conducted, which examined definitions, characteristics, preconditions, outcomes, and boundaries on the state of AI in nursing. A total of 18 quantitative, qualitative, mixed-methods, and reviews related to AI in nursing were retrieved from the CINAHL and EMBASE databases using a Boolean search. Presently, the concept of AI in nursing is immature. The characteristics and preconditions of the use of AI in nursing are mixed between and within each other. The preconditions and outcomes on the use of AI in nursing are diverse and indiscriminately reported. As for boundaries, they can be more distinguished between robots, sensors, and clinical decision support systems, but these lines can become more blurred in the future. As of 2021, the use of AI in nursing holds much promise for the profession, but conceptual and theoretical issues remain.

## Introduction and background

The world has been continuously experiencing social, economic, political, cultural, and technological change, and one that is anticipated to transform all aspects of society, including science in itself, is artificial intelligence (AI). AI is currently making worldwide headlines, being a significant influence in almost every field, and healthcare is no exception. Therefore, it is without surprise that this AI revolution is being reflected upon in the literature, with a 45% increase in the number of peer-reviewed healthcare AI publications within the last five years [1]. Current definitions of AI in healthcare vary, and AI has multiple applications including optimizing electronic medical records (EMR), virtual patient education, geocoding health data, social media analysis, epidemic and syndromic surveillance, predictive modeling, integration with mobile health (i.e., heart rate monitoring and analysis), and analyzing medical imaging [2]. Furthermore, the use of AI in healthcare, like how it is being rolled out in society, is likely going to affect all aspects of it, with a significant amount of development anticipated to occur in cutting-edge healthcare innovations. For example, the extraordinary analytic ability of AI allows for the capability to take into consideration individual traits, such as the genetic data of the patient, to deliver targeted treatments, paving the way towards personalized medicine [3]. Thus, AI in healthcare enhances existing systems, delivers its own form of healthcare, and accelerates and transforms healthcare innovations and discoveries.

The use of AI has moved beyond the abstract and has materialized in a select few nursing contexts. Currently, there are no formal evidence syntheses (i.e. scoping reviews or systematic reviews) or authoritative publications in the literature regarding the use of AI in nursing, with the present literature landscape being limited to empirical articles, opinion pieces, and literature reviews of limited scope. Nonetheless, even among the few existing nurse-centric AI articles, there is tremendous diversity, with examples being using AI to predicting readmissions in diabetic patients [4], evaluating nurse job performance [5], simulating nurses as a chatbot [6], and improving nursing documentation through autocomplete functionality [7]. Thus, the use of AI has an unprecedented potential of influencing almost every aspect of nursing care, and we are likely to see even more applications soon.

Nursing gap

Nurses, being the largest proportion of healthcare workers, would theoretically use and benefit the most from AI technologies. However, the overall adoption of new technologies by nurses is quite slow, and the use of AI in nursing is considered to be in its infancy [8]. Presently, there is a gap in the development and deployment of AI between nursing and medicine. This is evidenced by the fact that there are much fewer articles on the topic of nursing AI and no evidence syntheses. This is sharply contrasted with the multitude of available literature [9] and dedicated academic journals, such as Artificial Intelligence in Medicine. Although it is understandable that AI has several physician-oriented applications, such as the diagnosis and interpretation of medical imaging, there are still several key healthcare AI technologies that can be leveraged by nurses. Thus, even though nurses are an integral part of the healthcare team’s decision-making processes, they are often excluded from technological development, which can lead to suboptimal deployment and scaling of healthcare AIs.

One reason for this discrepancy is due to the technologically averse nature of many nurses [10], which is further exacerbated by negative popular portrayals of AI and technology within the media, with AI often being dubbed as “monsters” or “terminators” [11]. As a result of this adversity and overall negative attitude towards technology and especially AI, there is a general lack of understanding of the benefits, challenges, and long-term implications from nurse clinicians, researchers, and theorists. On the clinician-researcher angle, this translates to less research interest in nursing AI, leading to a limited understanding of nursing AI knowledge, cognitions, attitudes, routines, social influences, organization, and resources, contributing to the overall suboptimal implementation in practice [12]. Relatedly, the lack of empirical evidence and authoritative voices created a space where definitions of AI are used interchangeably, namely AI, robots, machine learning, and neural networks [13]. In the few existing publications on the use of nurse-centric AI, some of them use extremely technical language [14-16], likely due to the dominance of the computer scientists within the research team. Thus, for AI to realize its full potential in nursing, a first step is to standardize and clarify the various terms and definitions.

Concept analysis

Language is an essential component of the human experience and serves as the sole medium to communicate our thoughts and ideas. Concepts can be formally defined as “a complex mental formulation of empirical experience” [17]. The examination and analysis of concepts have the potential to determine the state of the science, clarifying what is known of the concept and used as a first step in enhancing the knowledge base of the discipline [18]. Thus, concepts form the theoretical realm of a discipline, and the advancement of the discipline relies on the continuous development of these concepts [19]. As the use of AI in nursing is in its infancy, conducting a concept analysis would be a useful first step and set the foundation for future knowledge development in this new area.

Although a concept analysis has not been conducted on the use of AI in nursing, a few relatable and relevant concept analyses have been conducted. Tacy et al. [20] conducted an analysis on the concept of technostress using the method outlined by Walker and Avant [21]. This concept analysis portrayed an excellent picture of technostress and its potential ubiquity within the modern nursing context, including addressing computer-related stress and the fear of using technology. Nonetheless, in the article, there is no mention of AI, and the author overall fails to capture the evolving role of technology. In another concept analysis, Paige et al. [22] conducted a concept analysis on eHealth literacy, also using the method outlined by Walker and Avant [21]. Although the revealed challenges and incongruities of eHealth literacy largely mirror that of AI, again there is no mention of it and the impact of AI on eHealth. The reason why the use of AI in nursing has not been addressed in these concept analyses is its relatively recent emergence in the literature and healthcare settings. Therefore, to set the foundations for nursing AI knowledge development, the purpose of this concept analysis is to clarify the conceptual components of AI in nursing and to determine its conceptual maturity.

## Review

Methods

The procedure used for this concept analysis followed the criteria set by Morse et al. [19] for concept evaluation, allowing for the examination of the definitions, characteristics, preconditions, outcomes, boundaries, with all leading to the discovery of the maturity of the concept. Although there are other methods of conducting concept analyses, the approach outlined by Morse et al. [19] is appropriate for this context, as it allows concepts to be evaluated with regards to clear boundaries. These boundaries are necessary, as currently there are no clear definitions as to what nursing AI is, with numerous terms being used interchangeably. Therefore, there needs to be a clear clarification first, before any further knowledge development or theorization can be done. Furthermore, the use of AI in nursing is at the intersections between nursing, medicine, and computer science, making it a highly multidisciplinary concept. This is congruent with the recommendations laid out by Morse et al. [19], who suggest theorists to not limit their literature review to a single discipline. However, one guideline by Morse [23] will not be followed word to word, as the literature of AI in nursing is currently nebulous, present with multiple and interchangeable definitions, and is not well understood, thereby necessitating a mix between the so-called concept development, delineation, and comparison. The lack of a comprehensive definition for AI in nursing is troubling, and future researchers and theorists run the risk of adopting a standard dictionary AI definition as the basis of their analysis [19]. This risk leads to even greater implications for the concept of AI in nursing, as there are multiple competing definitions. As there are a variety of terms and definitions within the AI nursing literature, it is important to clarify and delineate these definitions, of which Morse’s approach is best suited. Therefore, to address these overarching issues, a concept analysis was conducted following Morse’s methods.

Sampling

A search was conducted in December 2020 on the CINAHL and EMBASE databases. Only one database was chosen because of time and resource limitations. CINAHL was chosen because of its emphasis on journal articles related to nursing, allied health, and healthcare, rather than experimental, technical, and engineering articles. EMBASE was chosen because of its complementary emphasis on medicine and biomedical research. Inclusion criteria include studies published within the last five years (2015-2020), written in English, peer-reviewed, and containing the use of AI for nursing as a primary topic. The use of AI in nursing is deemed to be a primary topic if there is at least one nurse as a co-author, the end-users involve solely or a significant (>50%) number of nurses, and the purpose of the AI is designed to assist in a task that is relevant to nurses. Exclusion criteria include opinion articles, letters, editorials, and conference proceedings. The choice of having a five-year time limit was due to the short timeframe of this assignment, in addition to the fact that AI technology is evolving at an exponential rate, and any AI nursing innovations from more than five years ago are likely to be not in use or outdated. The major headings used to search CINAHL are outlined in Table 1. The subject headings used to search EMBASE can be found in Table 2. The initial search of both databases resulted in a total of 102 articles, of which the titles and abstracts were screened. A resultant 18 full texts (11 quantitative, three qualitative, two mixed-methods, and two literature reviews) were selected, read, and imported into NVivo 12 for data analysis. The articles and summary of their major characteristics are outlined in Table 3.

**Table 1:** CINAHL Search Strategy

Nursing	AI
Nurses	Artificial Intelligence
Nursing Knowledge	Machine Learning
Nursing Practice	Neural Networks (Computer)
Education, Nursing	
Nursing Role	
Nursing Care	
Nursing Leaders	

**Table 2:** EMBASE Search Strategy

Nursing	AI
Nurse	Artificial intelligence
Nursing	Machine learning
Nursing as a profession	Artificial neural network
Nursing staff	
Nursing knowledge	
Nursing research	
Nursing care	
Nursing role	
Nursing process	
Nursing education	
Nursing practice	
Nursing management	

**Table 3:** Characteristics of Included Studies

Author	Year	Study Design	Country	Summary
Kwon et al. [4]	2019	Quantitative	Canada	Use of machine learning to predict readmission in diabetic patients
Khanjankhani et al. [5]	2017	Quantitative	Iran	Use of artificial neural network to predict nurses' job performance
Shorey et al. [6]	2019	Quantitative	Singapore	Use of virtual patients (AI) to develop communication skills in nursing students
Greenbaum et al. [7]	2019	Mixed Methods	USA	Use of machine learning to reduce redundancy in nursing documentation
Bose et al. [14]	2019	Quantitative	USA	Use of machine learning to identify critical elements in nursing documentation
Li et al. [15]	2019	Quantitative	Republic of China (Taiwan)	Use of AI to predict pressure injuries in end-of-life patients
Ladstätter et al. [16]	2016	Quantitative	People's Republic of China	Use of artificial neural network to model burnout in nurses
Cooper et al. [24]	2021	Quantitative	USA	Use of AI to identify patients at risk of sepsis
Hernandez [25]	2019	Review	Saudi Arabia	Theoritization on the use of "nursing chatbots"
Cato et al. [26]	2020	Review	USA	Implications of AI for nurse leaders
Fritz and Dermody [27]	2019	Mixed Methods	USA	Use of AI sensor technology for remote patient monitoring in-home care
Barrera et al. [28]	2020	Qualitative	UK	Experiences of using an AI for remote psychiatric patient monitoring
Brom et al. [29]	2020	Quantitative	USA	Use of machine learning to identify patients at risk of readmission within 30 days
Griner et al. [30]	2020	Quantitative	USA	Use of AI to forecast census and capacity flow of patient admissions
Gonçalves et al. [31]	2020	Qualitative	Brazil	Experiences of nurses using an AI to identify patients at risk of sepsis
Beauchet et al. [32]	2018	Quantitative	France	Use of artificial neural network to predict falls in inpatient older adults
Clavelle et al. [33]	2019	Qualitative	USA	Use of AI to qualitatively analyze definitions of nursing care
Zachariah et al. [34]	2020	Quantitative	USA	Use of AI to predict urinary tract infections during hospitalization

Data Analysis

The data were analyzed through an inductive content analysis method guided by the approach of Elo and Kyngäs [35]. The aim of content analysis is to achieve a condensed and broad description of the concept and involves open coding, creating categories, and abstraction [35]. The inductive approach is recommended when there is little or fragmented knowledge about the concept [35] and is also strongly advocated by Morse et al. [36], who cautions readers to avoid the “deductive trap”. This is necessary for the literature in AI nursing, as the research is currently fragmented across definitions (i.e., robot versus neural network), purposes (i.e., used to predict patient outcomes versus assisting in qualitative data analysis), and authorship (majority nurses versus mixed computer scientists and nurses). However, the inductive approach to data analysis appears counterintuitive to the concept analysis method detailed by Morse et al. [36], which evaluates a concept across very delineated components, namely its definitions, characteristics, preconditions, outcomes, boundaries, and maturity. In order to ensure analytical clarity, I have decided to undergo the inductive approach while respecting the delineations between the components detailed by Morse, allowing for induction to occur within and across each category.

Results

Definitions

There are numerous existing definitions of AI, and there is no standardization (every article uses its own definition of AI). In addition, the term “nursing AI” is not explicitly defined in any of the articles. The term AI itself can be defined in two manners; simplistic and complex. A simplistic definition of AI can be “computers mimicking human behavior” [24]. A more complex definition of AI is “multiple technologies that can augment human activities in the form of machine learning, to process and learn with raw data and deep learning, [sic] to stimulate decision-making using complex artificial neural networks” [25]. Definitions are currently scattered with only three articles formally defining AI [24,26,27], six not offering any formal definition [6,7,25,28-30], three defining only machine learning [4,14,31], three defining only neural networks [5,16,32], and one defining only decision trees [15]. Therefore, the definitions of AI used in the literature include machine learning, neural networks, and decision trees.

Machine learning is the most common definition of AI and is mentioned in 13 of the 18 articles. One definition of machine learning that is not too simple or complex is by Gonçalves et al. [31], who describes machine learning as “the science of getting computers to learn and act like humans do, and improve their learning over time in an autonomous way, by feeding them data and information in the form of observations and real-world interactions”. The second most common definition of AI is neural networks, which are mentioned in eight articles. Neural networks are defined by Khanjankhani et al. [5] as an application of AI derived from mathematical models of the brain, used for forecasting, and meant to detect complex nonlinear relationships between dependent and independent variables. Lastly, there are decision trees, which are mentioned in six articles. Decision trees are the most poorly defined, and any attempts to define decision trees are filled with computer science jargon. The most comprehensible definition is one by Li et al. [15], who define decision trees as a mathematical model, which “implements a top down divide-and-conquer method that recursively partitions a dataset into smaller subdivisions. These procedures are the basis of a set of tests defined at each branch in the tree”.

Overall, machine learning, neural networks, and decision trees are seen as applications of AI, which is the global term covering machines mimicking human intelligence. The explanations and elaborations provided for each of these definitions become progressively more complex and mathematical (from machine learning to neural networks to decision trees).

Characteristics

The characteristics of the use of AI nursing are algorithms, continuous development, improving human processes, and nursing voice. Although all these characteristics are necessary for the use of AI in nursing, there are varying degrees of emphasis within the 18 articles, including some articles that do not explicitly describe every characteristic. One major exclusion is robotics, as it is related largely to hardware, but it can have some overlap with AI. This is further discussed in the "Boundaries" section.

The first characteristic, algorithms, is ubiquitous and mentioned within every single article. Algorithms are typically sophisticated statistics programs capable of instantly solving computations so large that it would take a human years to complete [27], with the overarching goal of making accurate predictions [31]. However, no algorithm is the same, and each nursing issue would require a new or even multiple new algorithms. Nonetheless, all algorithms involve the use of complex data and analysis of trends [30], which allow for the application of an enormous number of observations and predictors [4,16]. Despite its impressive abilities, algorithms are still limited by the data and method of input, and all algorithms require a form of input [4]. Data entry can come either automatically through EMRs [26] or manual human entry.

Even if an algorithm appears to appropriately analyze and predict the nursing issue at hand, it would still require continuous development. Continuous development is necessary, as the real world can never be truly quantified as a finite number of variables interacting with each other. Thus, development never ends, as long as the AI is in use within a nursing context and all require careful evaluation and implementation [28]. In addition, there is a need to continuously quantitatively test the algorithm, to ensure that there are no errors, and to compare between competing AIs [16]. After an AI has been developed and tested, with appropriate ongoing evaluation plans in place, it still needs to demonstrate its ability to improve human processes. This would involve enhancing decision-making, such as identifying important aspects [14,31] and prioritization. Overall, by improving human processes, AIs are expected to reduce the time wasted and mental computations that are required of nurses [24].

All the aforementioned characteristics are relevant for any AI implemented within a healthcare setting. However, for it to be nursing-specific, there needs to be a nursing voice throughout all phases of development and evaluation. No algorithm can capture an infinite number of variables; therefore, it is up to the nurses to decide which ones are important, ensuring that the AI is shaped by nurses’ unique knowledge [4]. Besides being part of the AI development, nurses are also the end-users, and the AI should allow nurses to form a complementary partnership with it. Specifically, AIs are trained in data recognition but cannot intuitively understand or incorporate the context [27]. Therefore, it is important to promote the integration of the AI’s quantitative thinking with the nurse’s critical thinking [30].

Preconditions

Preconditions of the use of AI in nursing include conditions that must be present for the use of AI in nursing to develop and the circumstances that influence the manifestation of it. Conditions that must be present for the use of AI in nursing include data availability, interdisciplinary collaboration, and underlying issues. Circumstances that significantly influence the development of nursing AIs, but are not necessarily essential, include technological innovation and nurse engagement.

One of the most important preconditions for any AI is the availability of data. All AIs require pre-existing data to build and train the AI, and the data should ideally be continuously given to update the AI. The data used for nursing AIs are incredibly varied, from qualitative data of nursing performance [33], to nursing notes, medication administration records, and nursing flow sheets [26]. Typically, data comes from EMRs, which allow for instant capture of data [31]. Also, if the AI uses data from a standardized EMR, it can allow for the AI to be used across multiple healthcare systems [29].

Another crucial precondition is the collaboration between nurses, computer scientists, software engineers, and information technologists. Nearly all the articles have authors or acknowledge people who come from a technical and non-nursing background. This collaboration should also be done as early as possible, using jargon-free language from both sides. This would nurture a cohesive team environment for members of vastly different disciplines, prevent miscommunications, and ensure shared goal accomplishment [30]. Furthermore, this collaboration goes both ways, as the computer scientists can consult nurses to ensure clinical accuracy, while nurses can consult the computer scientists to troubleshoot and propose adjustments to the AI [30].

For a nursing AI to develop, there needs to be an underlying issue within the nursing context. In other words, a nurse-centric AI is a tool, and if there is no ongoing problem, then there is no need to create or use that tool. Examples of issues that would necessitate an AI could be the need to study nursing burnout [16], lack of patient actors for clinical education [6], and a general lack of time or resources [14]. In addition, AIs are developed to address and predict the occurrence of clinical issues, such as pressure ulcers [15], urinary tract infections [34], and sepsis [24]. These clinical issues are typically complex and can be caused by a multitude of factors, which is why an AI can enhance the nurse clinical decision-making process.

Next, technological innovation significantly influences the development of the AI. More technology means more computing power and better algorithms [26]. Nursing AIs can benefit from recent products from technology companies, such as when Shorey et al. [6] modified Google’s AI chatbot into a nursing one. Lastly, there needs to be nurse engagement, which can be described as nurse interest, training, and technological competence. These factors of nursing engagement are all interrelated, as the more the nurses train, the more familiar they become with the AI and its technological language over time [7]. All these factors can be enhanced by the presence of nurse champions, who can promote the AI not only with the nurses but also with the other members of the healthcare team [31].

Outcomes

There are numerous outcomes as a result of the use of AI in nursing, namely on the patient, nurse, and organization level. However, there is no AI with significant and direct outcomes at each level. Normally, an AI would be developed and targeted at either the patient or nurse level, and both have indirect organizational-level outcomes.

For the patient level, the main outcome is increased awareness of patient conditions. Usually, this would involve prediction, such as determining which patient is most at risk for falls [32] or using the EMR to assess which discharged patients are most likely to be readmitted [29]. This increased awareness of patient issues results in tangible patient outcomes, such as faster identification of patient deterioration, leading to a decreased patient mortality rate [24]. Despite promising patient outcomes for many AIs, a few studies [4,28,32] also show mixed patient outcomes, but none of the nursing AIs lead to negative outcomes. Interestingly, patient privacy does not appear to be compromised through the use of nursing AIs, with one study by Fritz and Dermody [27] observing an increase in patient privacy. Fritz and Dermody [27] evaluated the use of home sensors and remote patient monitoring by an AI and noted that the sense of privacy increased because there are less in-person visits by the nurse.

The outcomes at the nurse level are closely related to patient-level outcomes. The most noticeable and common outcome is increased performance, which can be through increased decision-making and collaboration with other members of the healthcare team. Also, both decision-making and collaboration are related, as the predictions of the AI can be discussed as a team, leading to team learning and decision-making [30]. Generally, a nursing AI is meant to reduce the burden, increase the nurse’s efficiency, and save time. In clinical settings, this can translate to less documentation [7] and having instant notifications of patient deterioration [24]. Nonetheless, an AI can also be designed without directly considering patients, such as an algorithm to predict a nurse’s job performance [5] or a virtual patient for nursing education [6].

For the organizational level, efficiency is key, and it can be accomplished through the identification of care priorities [14], optimization of staffing and patient bed allocation [30], and prediction of nurses’ job performance [5]. Several staffing issues can also be identified by the AI, including job stressors [16] and priorities described by nurses [33]. Also, the positive outcomes from both the patient level (i.e. reduced patient mortality) and staff (i.e. more documentation time saved) level result in greater organizational efficiency and significant cost savings [24].

Boundaries

Robots are considered the most significant boundary of AI. Within the sample of 18 articles, robots are barely mentioned due to no major or subject headings related to “robots” or “robotics” being used during the literature search. Nonetheless, robots and/or robotics are still mentioned in four articles. The use of a “robot” is the primary topic of Gonçalves et al. [31], who calls their algorithm to detect sepsis a “robot”. This is contrasted with the application of the term “robot” in other articles, as robots are meant to replace a mechanical motion or repetitive action of a nurse [6]. Therefore, robots are considered outcomes of the field of robotics and can be applied to the field of healthcare [16], creating a “humanoid” robot [25]. One excellent example of the application of a robot is the virtual patient developed by Shorey et al. [6]. Specifically, in the study by Shorey et al. [6], the predictive and reactive algorithm determining the speech of the virtual patient can be construed as part of the AI, while the computer-generated model of a human patient can be seen as the robot.

Another boundary is with sensors, which are mentioned in three articles. Similar to the boundary of robots, sensors do not engage in any predictions or calculations, but they can be integrated with AIs, which is the focus of two of the three articles [27,28]. Thus, sensors are the means by which data are gathered, which will be subsequently analyzed by the AI. Sensor technology is incredibly diverse, as it can be infrared [28] or motion-detecting cameras and contact (i.e., to detect a person on a chair or bed), light, temperature, and humidity sensors [27]. Naturally, the more advanced a sensor is, such as the infrared sensor used by Barrera et al. [28] which can detect multiple items, including the respiration rate of the person, the more sophisticated the AI will have to be to integrate this complex information. This is contrasted with robots, as robotic technology is arguably less developed than sensor technology and currently only serves as an interface or physical model of the AI. Nonetheless, in the future, there may be an incorporation of sensor, robotic, and AI technology into one comprehensive unit.

Lastly, clinical decision support systems (CDSSs) are mentioned by Cato et al. [26] and can be considered as the final boundary. CDSSs are defined as a system that provides clinicians with computer-generated clinical knowledge and patient-related information that is intelligently filtered and appropriately presented [26]. Although Cato et al. [26] construed AI as being the “fuel” to the “engine” that is CDSS, the reality is that AI is much more broader than that. This is because AI is not limited to enhancing clinical decision-making, and it can be used in a multitude of different contexts.

Discussion

Conceptual Maturity

In order to operationalize the concept of the use of AI in nursing in theory, research, and practice, it is necessary to evaluate the maturity of a concept [19]. The conceptual evaluation begins by assessing if the concept is well-defined, in which a well-defined concept has consistent definitions, while poorly developed concepts will have definitions that are hard to find or that inadequately describe the phenomenon [19]. Also, the characteristics, preconditions, and outcomes will be less identified, and the boundaries will be unknown [19]. This is currently the case for the concept of the use of AI in nursing.

To begin, no studies have explicitly formulated a definition of a “nursing AI” or “the use of AI in nursing”. Although Robert [13] did detail the state of AI in nursing, it is largely a broad overview rather than a rich conceptualization. Furthermore, this is complicated by the fact that not even “AI” is consistently defined, and numerous competing definitions exist, including machine learning, neural networks, and decision trees. Although there is a sense of coherence, where it is agreed upon that those previously mentioned terms are under the overarching field of AI, this hierarchy is not strictly enforced, and these terms can be presented as their own. Also, even if the authors do define AI, many do not even define what machine learning, neural networks, or decision trees are. This lack of definition clarity has severe implications for research, practice, and theory, as it limits the readership to only those with a significant amount of computer science knowledge, creating a knowledge divide.

Currently, the characteristics and preconditions of the use of AI in nursing are mixed between and within each other. The most prominent example of mixing is the “nursing voice” characteristic, which significantly overlaps with the preconditions of collaboration and nursing engagement. Since the topic of this concept analysis is the use of AI in nursing, not just the use of AI, the nursing voice is a definite characteristic. But AI also exists as an application outside of the nursing context, which is why nurses are needed to be part of the development team. Furthermore, a nursing AI can be developed, but its purpose comes from the patient, nurse, or organizational outcomes, not from the fact that it was simply created. This is further complicated by the ambiguous development timeline (no certain timeframe for when an AI is “finalized”) and need for continuous nursing AI evaluation. Thus, coupled with the precondition of nurse engagement, it is crucial for a nursing AI to have a successful implementation and long-term sustainability. Nonetheless, if it is necessary to evaluate the “strength” of the delineations, then collaboration would be most important as a precondition, since it is important to begin with a nurse-centric AI. If AIs are not developed with nurses in mind, then it would set a dangerous precedent for the future of nursing AIs, as the state of this concept and field is still in its infancy. In general, the delineations between and within the characteristics and preconditions are unclear, contributing to the conceptual immaturity of the use of AI in nursing.

As of the writing of this concept analysis, there are no universal outcomes associated with the use of AI in nursing. Even though organizational outcomes arise from nearly every AI, these are not direct outcomes, and they are difficult to predict, examine, and interpret. Also, collaboration, which can be between nurses and the healthcare team or nurses and the computer scientists, is another prominent outcome of the use of nursing AI. This collaboration is related to the nursing voice characteristic and nurse engagement precondition, and the margins separating them are not delineated. Also, it is presently unclear whether the creation of a nurse-centric AI is an outcome or if there needs to be tangible patient, nurse, or organizational outcomes. If going by the latter definition, then almost no nursing AI studies will have any outcomes. This can be because of the scarcity of nursing AI literature and the fact that long-term or complex outcomes cannot be observed within this short time frame.

Lastly, there is a clear violation of the robot and CDSS boundaries, with the sensor boundary remaining largely intact. One prominent example is by Gonçalves et al. [31], who call their AI a robot. This is also an opinion voiced in an article by Robert [13], where robots are considered to be part of AI. However, it is clear that this is likely not the case, although there can be areas of overlap, such as through virtual patients or a robot equipped with AI capabilities. According to this concept analysis, that application would be incorrect, as robots are meant to replace a mechanical motion or repetitive action. If the AI uses algorithms to deliver patient, nurse, or organizational outcomes, then it should not be considered as a robot. However, the differences between the terms robot and AI may cause some confusion among nurses and authors who are unfamiliar with the topic. Furthermore, the use of these terms in other languages and cultures is unclear, and it might very well be the case for Gonçalves et al. [31], as the authors come from Brazil. The use of sensors is a clearer boundary, perhaps due to the fact that sensor technology is more established within the healthcare field [37]. But this would require a reassessment when advanced AIs are developed in the future, which will incorporate AI, robot, and sensor technology into one. Also, the relationship between CDSS and AI is presently unclear, and there is only one study that mentions it within the 18 articles. Since CDSS can involve non-AI technologies, and that AI can be applied in other domains besides clinical decision-making, it would be advisable to avoid grouping AI with CDSS. However, both fields are in their infancy, and perhaps in the future, they may converge.

Overall, due to the presence of competing definitions, lack of definition clarity, confusing interactions between characteristics, preconditions and outcomes, and semi-undefined boundaries, it can be concluded that this concept is presently immature. Future concept analyses should continue to use the criteria set out by Morse et al. [19], as the state of the literature and technology is rapidly evolving, and the results from this concept analysis may not be relevant in a few years.

Limitations

This concept analysis has its limitations. One limitation is the relatively small number of studies included in the analysis. Future concept analyses could be conducted using a larger number of databases and guidance from a professional librarian. Relatedly, only subject headings were used in the search strategies, and the inclusion of keywords could have enriched the sample. Another limitation is the exclusion of robotics and CDSS literature, as their inclusion would likely make the boundaries clearer and more backed by the literature. However, the relationship between these different but complementary concepts is presently unclear and can be a direction for future concept analyses. Lastly, the state of AI is rapidly changing, and findings from this concept analysis might not apply to future practice, as new characteristics, preconditions, and outcomes emerge.

## Conclusions

This is the first concept analysis conducted on the use of AI in nursing and therefore sets the foundations for further research, knowledge synthesis, and theorizing. Furthermore, this concept analysis highlights the importance of differentiating robots, CDSS, and AI, and proposes the evaluation of nursing AIs according to its patient, nurse, and organizational-level outcomes. Relevantly, there are no universal outcomes associated with the use of AI in nursing, and the clearest boundary is between AI and robots, though CDSS can sometimes be considered a subset of AI. Even though organizational outcomes arise from nearly every AI, these are not direct outcomes, and they are difficult to predict, examine, and interpret. The characteristics and preconditions of the use of AI in nursing are also mixed between and within each other. Presently, the use of AI in nursing holds much promise for the profession, but conceptual and theoretical issues remain. Following this, it is determined that the concept of the use of AI in nursing is presently immature. This concept analysis also introduced and presented standardized definitions, which can be used for future research and analyses. Future concept analyses in this subject should also be conducted using Morse’s approach, until it is considered to be more mature.
